# Outdoor Walking Speeds of Apparently Healthy Adults: A Systematic Review and Meta-analysis

**DOI:** 10.1007/s40279-020-01351-3

**Published:** 2020-10-08

**Authors:** Elaine M. Murtagh, Jacqueline L. Mair, Elroy Aguiar, Catrine Tudor-Locke, Marie H. Murphy

**Affiliations:** 1 University of Limerick, Limerick, Ireland; 2 University of the West of Scotland, Glasgow, UK; 3 University of Alabama, Tuscaloosa, USA; 4 University of North Carolina at Charlotte, Charlotte, USA; 5 Ulster University, Newtownabbey, UK

## Abstract

**Background:**

Walking outdoors can be used by many individuals to meet public health guidelines for moderate-to-vigorous-intensity physical activity. The speed at which adults walk may be a proxy for intensity. Traditional estimates of indoor walking speed are unlikely to reflect self-selected usual or other instructed paces of outdoor walking speed.

**Objective:**

To inform estimates of pace-based walking speed of apparently healthy adults in outdoor settings.

**Methods:**

We searched four electronic databases for articles published in English between January 1970 and March 2019. Studies that reported walking speed (m/s), cadence (steps/min), or intensity (mL/kg/min) of ambulatory, apparently healthy, and community-dwelling adults (> 18 years) were included. Walking speed categories were defined according to the description provided in each study. Meta-analysis was used to synthesise speed, cadence, and intensity data by slow, usual, medium, fast, and maximal pace (where reported).

**Results:**

Thirty-five studies, representing 14,015 participants (6808 women, 5135 men, and 2072 sex not specified), were identified. The mean (95% CI) walking speed for slow, usual, medium, fast, and maximal pace was 0.82 (0.77–0.86), 1.31 (1.27–1.35), 1.47 (1.44–1.49), 1.72 (1.64–1.81), and 1.62 (1.45–1.79) m/s, respectively. Mean cadence (95% CI) for usual and fast paces were 116.65 (114.95–118.35) and 126.75 (121.87–131.63) steps/min, respectively. The mean oxygen consumption (95% CI) for the usual and medium paces was 11.97 (11.69–12.25) and 13.34 (12.94–13.73) mL/kg/min, respectively**.**

**Conclusion:**

These findings provide greater clarity with regard to how various indicators of enacted walking pace, speed, and intensity overlap and how each can be best communicated in the real-world setting to optimise health-related outcomes. Pace-based instructions can be used to support walking in outdoor settings within public health guidelines.

**Electronic supplementary material:**

The online version of this article (10.1007/s40279-020-01351-3) contains supplementary material, which is available to authorized users.

## Key Points

**Table:** 

We reviewed studies that measured walking speed of apparently healthy adults in outdoor settings.
We provide expected values for speed, cadence, percent maximal heart rate, and oxygen consumption for slow through maximal paced walking.
Walking outdoors at a usual pace was associated with an average speed of 1.31 m/s, a cadence of 116.65 steps/min, and an oxygen consumption of 11.97 mL/kg/min, meeting/exceeding public health thresholds.

## Introduction

Walking is the most commonly reported exercise among adults [1]. Walking demands little skill, facility, or equipment requirements and is socially acceptable for most individuals across most cultures worldwide making it the near-perfect form of exercise [2]. Outside of purposeful exercise, walking is also commonly performed in the course of daily personal transport/commuting, recreation, or domestic/occupational activities. Given the dose–response relationship between physical activity and health and the disproportionate population health gains derived from encouraging the most inactive to increase activity [3], walking has become the cornerstone of physical activity promotion for public health and the gateway through which inactive and low active individuals can initiate access to these benefits.

Current physical activity guidelines recommend that adults accumulate at least 150 min of moderate-intensity physical activity each week [4]. As implied, the health benefits of walking depend, in part, on its intensity [5]. Although walking volume metrics (time, distance, and/or accumulated steps) have become common parlance in health promotion, there is less clarity regarding accessible expressions of intensity (traditionally expressed in physiological terms as rate of oxygen consumed or mL/kg/min relative to an individual’s maximum). Alternatively, walking speed may be a proxy for intensity. Irrespective of level of personal fitness, walking at a faster speed results in a higher relative exercise intensity and, therefore, presents a greater stimulus for health benefit. It has been repeatedly demonstrated that walking speed is a stronger predictor of risk than volume in terms of all-cause mortality, heart failure, and disease risk, across a continuum of volumes [6, 7]. Speed-based intensity recommendations have been derived primarily from laboratory studies; the only published meta-analysis of walking speed did not distinguish between indoor and outdoor settings [8]. Walking speed thresholds determined in clinical settings have been used to classify walking independence [9], as part of geriatric assessment [10], and as a summary indicator of frailty [11]. Limitations of assessing walking speed in the clinical setting include measurement noise, bias due to brevity, and variability due to participants’ motivation and learning effect [12]. Thus, controlled walking in the laboratory setting is not representative of pace-based speeds enacted during the course of daily living. Determining the speed individuals choose to walk in different contexts or in response to specific instructions, and the physiological demands of these self-selected paces, may help those promoting walking to ensure that advice on walking pace is likely to result in moderate to vigorous physical activity and, therefore, contribute to meeting current PA guidelines. The primary objective of this systematic review and meta-analysis was to inform estimates of pace-based walking speed of apparently healthy adults in outdoor settings.

## Methods

The protocol for this systematic review and meta-analysis was registered with PROSPERO: International prospective register of systematic reviews (registration number CRD42017051911) [13].

### Inclusion Criteria

We included observational, randomised-controlled trials and pre-post intervention studies involving ambulatory, apparently healthy (free-living adults without a clinical diagnosis of disease), community-dwelling adults (> 18 years of age). All walking assessments were conducted outdoors (i.e., not on a treadmill or in any other controlled indoor space). Studies were excluded if they focused exclusively on adults with lower limb conditions, musculoskeletal conditions, or gait issues that may have impaired walking ability, in-hospital patients, or clinical populations. If a study included an experimental group involving a clinical population and a “healthy” control group, the study was incorporated to allow inclusion of data collected from the control group.

The primary outcome of interest was pace-based outdoor walking speed, either freely chosen or in response to a verbal instruction, measured over a distance of at least 3 m (10 feet). Studies were included if they reported speed in quantitative units (e.g., m/s, km/h). Studies were excluded if the walking protocol involved an abrupt change of direction. Secondary outcomes included any other direct or indirect indicator of intensity (e.g., cadence, metabolic equivalents (METs), percent maximal heart rate (HR_max_), percent maximal aerobic capacity, and energy expenditure).

### Search and Selection

We searched the following electronic databases for English language articles published between January 1970 and March 2019: OVID (Medline), CINAHL, SCOPUS, and Web of Science. We hand-searched reference lists of identified studies and systematic reviews to identify potentially relevant studies. The full electronic search strategy is presented in Electronic Supplementary Material Appendix S1.

We used online software Covidence to manage the study selection process [14]. Two authors (JM and EA) independently screened titles and abstracts to exclude records that did not meet the inclusion criteria for the review. A third author (EM) adjudicated any disagreements. The full-text versions of potentially eligible studies were then reviewed independently by at least two of the team of three authors (JM, EA, and EM). Disagreements were resolved through consensus. We collated multiple reports of the same study and treated one unique parent study as the unit of interest.

### Data Extraction

Data were extracted by one author (JM) using a pre-piloted data extraction form prepared using Microsoft Excel. A second author (EM) checked 20% of data for accuracy [15]. Disagreements were resolved by consensus. Extracted data included: (1) study setting (country, test location/surface); (2) participant characteristics (sex, age, height, body mass, and body mass index [BMI]); (3) method of measuring walking speed (test protocol); (4) walking pace category (description of pace provided in the original text); (5) walking speed (in originally reported units); (6) cadence, and (7) intensity (where and as reported). Study authors were contacted when specific data were missing or unclear [16–19]. The online software WebPlotDigitizer version 4.1 (https://automeris.io/WebPlotDigitizer) was used to extract data from a figure in one study [20].

### Quality Assessment

The NIH tool for Assessing the Quality of Observational Cohort and Cross-Sectional Studies was adapted to examine whether there was potential for bias in each study [21]. The following six questions were deemed directly relevant to our study purpose:

1. Was the research question or objective in this paper clearly stated?
2. Was the study population clearly specified and defined?
3. Was the participation rate of eligible persons at least 50%?
4. Were all the subjects selected or recruited from the same or similar populations (including the same time period)? Were inclusion and exclusion criteria for being in the study pre-specified and applied uniformly to all participants?
5. Was a sample size justification, power description, or variance and effect estimates provided?
6. Were the outcome measures (dependent variables) clearly defined, valid, reliable, and implemented consistently across all study participants?

Assessments were made independently by two authors (MM, CTL), with any disagreements resolved by a third author (EM). Assessors could select ‘yes’, ‘no’, or ‘other (cannot determine, not applicable, and not reported)’ for each question.

### Analysis and Synthesis

Walking speed was converted to m/s where necessary. For studies with several measurements of walking speed conducted at different time points (e.g., in the case of prospective studies), each measure was treated as a separate data point. The summary measures for all outcomes were mean and its associated standard deviation. Walking speed outcomes were categorised according to the description of walking pace provided in each study or the instruction given to participants (e.g., fast—“walk briskly” or “walk as fast as possible”). We collapsed synonymous pace terms into single categories based on the descriptions of pace that were provided in the original text; e.g., if a study described the pace as habitual/usual/normal/self-paced, it was assigned to the “usual category”. To avoid confusion between similar modifying terminology related to pace and intensity, we decided to reserve the adjective “medium” to describe pace (even if the original article described an instruction to “walk at a pace corresponding with moderate intensity”) and the adjective “moderate” to specifically describe intensity throughout.

Meta-analyses were conducted for each outcome of interest (walking speed, cadence, METs, percentage HR_max_, oxygen consumption, and energy expenditure). A minimum of three studies was deemed necessary to perform meta-analyses for each pace instruction/description category. The random-effects model was used as it allows for a greater level of natural heterogeneity between studies. Pooled results were reported as weighted mean with 95% confidence intervals. The I^2^ statistic was used to quantify the level of heterogeneity present. Pre-specified sub-group analysis of outcomes by sex was conducted where relevant data were available.

## Results

**Fig. 1:**
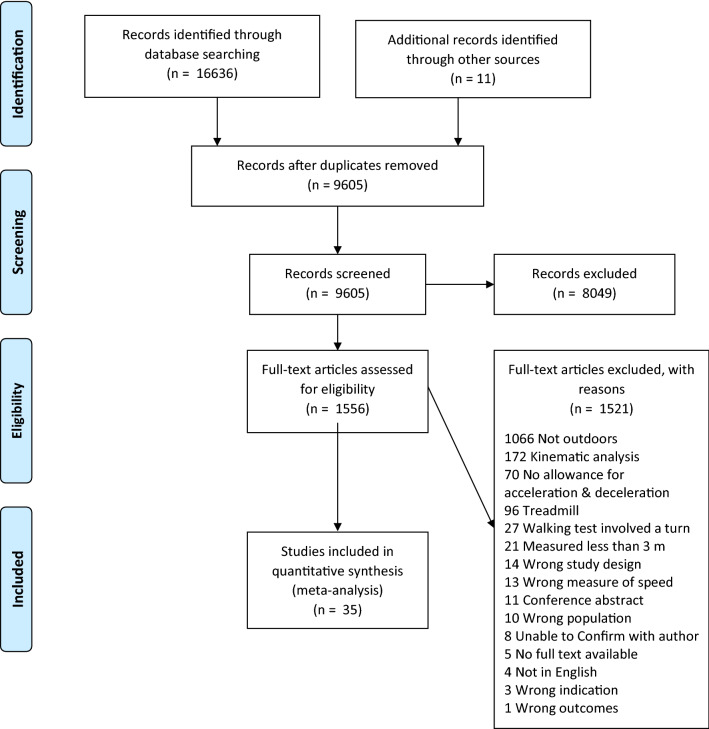
PRISMA flow diagram

Following removal of duplicates, a total of 9594 articles were identified by electronic searches and 11 additional articles from hand-searching strategies. After screening the title and abstract of the 9605 articles identified, 8049 were excluded as they did not meet inclusion criteria. The full-text versions of 1556 articles were then reviewed. Authors of 42 studies were contacted to confirm whether or not the measure of walking speed was conducted in an outdoor setting. Multiple reports of the same study were collated [22, 23]. Ultimately, 36 articles, representing 35 unique studies, were deemed eligible for inclusion. The reasons for exclusion at various stages of the search process are presented in Fig. 1.

The characteristics of identified studies are summarised in Table 1. All studies were observational in design. No randomised-controlled trials or pre-post intervention studies met the inclusion criteria. Studies were conducted in 13 countries, with the majority of studies conducted in Australia (*n* = 8), followed by USA (*n* = 7), and France and the UK (4 studies each). The identified studies represented 14,015 participants (6808 women, 5135 men, 2072 sex not specified), ranging in age from 18 to 90 years (mean = 44 ± 17 years). Mean BMI calculated from available data presented in 15 studies was 24.8 ± 3 kg/m^2^. The setting for measurement of outdoor walking speed varied across studies. The most frequently reported location was a path/track (20 studies). Other settings included: pavement/sidewalk (5 studies), athletics track (3 studies), grass (1 study), road intersection/crossing (2 studies), mixed terrain (2 studies), and unspecified outdoor settings (2 studies).

**Table 1:** Characteristics of included studies

Study	Country	Age (years)	Sex	Setting	Walking speed test protocol	Units
Abraham et al. [33]	France	Experiment 1: 32 ± 5 years Experiment 2: 21 ± 3 years	Experiment 1: M = 8 F = 2 Experiment 2: M = 5 F = 5	400 m outdoor synthetic track	Two different outdoor prescribed walking protocols with distances ranging from 50 to 400 m. The actual speed of each walking bout was calculated by dividing the distance by the time measured with a stopwatch	km/h
Ali et al. [34]	Pakistan	Range = 18–25	M = 12 F = 28	Even outdoor surface	Each participant was instructed to walk over a 20 m area selected by the physical therapist on an even outdoor surface at his or her normal pace wearing normal footwear. Gait velocity (m/s) was calculated by multiplying step length with cadence of each participant at the end of walk	m/min
Bargegol et al. [35]	Iran	NR	M = 359 F = 245	Two signalized and 2 un-signalized intersections in the city of Rasht	Using a stopwatch on the recorded videos for crossing time and speed of each pedestrian were calculated	m/s
Bargegol and Gilani [36]	Iran	NR	M = 3435 F = 3711	Two sidewalks in the city of Rasht	Sidewalks were filmed by video cameras	m/s
Bassey et al. [22]	UK	Range 66-NR	M = 56 F = 66	Paved outdoor course	Timed 100 m walk	km/h
Braham et al. [37]	Australia	Mean 34.7	M = 13 F = 36	An oval track of 130 m	The timed 50 m straight was used to determine walking speed. Smart Fusion timing gates (Smart Fusion, SmartSpeed, QLD, Australia) recorded at least two timed 50 m distances for each participant in all experiments, which allowed for calculation of speed in kilometres per hour	km/h
Brooks et al. [38]	Australia	Range 35–45 Mean = 40	M = 36 F = 36	Level, paved quadrangle	Mean walking speed was calculated for minutes 5–15 of walking. Structural beams evenly spaced around the quadrangle allowed us to estimate distance walked to the nearest 2.5 m	km/h
Caramia et al. [39]	Italy	Range = 21–23	M = 6 F = 4	Pedestrian walkway	200 m long straight path repeatedly walked until the end of the task sequence. The 20 central seconds of each task were used to extract 19 spatio-temporal gait parameters	m/s
Gates et al. [40]	USA	30 +	NR	Pedestrian intersections	Stopwatch was used to measure pedestrian crossing times (in person or video camera). The crosswalk lengths were measured	ft/s
Gunn et al. [17]	Australia	Range 35–45 Mean = 39.3	M = 12 F = 12	Paved quadrangle	Each subject received standardised instructions requesting them to walk at what they perceived to be a ‘moderate pace’ around a sheltered, level and paved quadrangle (141 m) for 13–15 min. [received from author]	km/h
Gunn et al. [41]	Australia	Range 35–45 Mean = 40	M = 36 F = 0	Level paved quadrangle	Each subject received standardised instructions requesting them to walk at what they perceived to be a ‘moderate pace’ around a sheltered, level and paved quadrangle (141 m) for 13–15 min. [received from author]	km/h
Gunn et al. [42]	Australia	Range 55–65 Mean = 60.6	M = 50	Covered level paved quadrangle	Each subject received standardised instructions requesting them to walk at what they perceived to be a ‘moderate pace’ around a sheltered, level and paved quadrangle (141 m) for 13–15 min. [received from author]	km/h
Hills et al. [20]	Australia	Obese group: Mean = 47.8 ± 10.8 Non-obese group: Mean = 36.9 ± 12.4	NR	Level 2 km grass track	Participants independently walked on a level 2-km grass track for more than 10 min	m/s
Le Faucher et al. [43]	France	Mean = 32 ± 14	NR	Outdoor athletic track (400 m long, flat area, free of compact trees, free of buildings, with just a gallery that runs parallel to one side of the track)	Six prescribed walking bouts of 2 min, 4 min, 30 s, 15 s, 1 min, and 8 min, separated with resting bouts of 30 s, 15 s, 4 min, 2 min, 1 min, and 8 min. Subjects walked at a freely chosen speed on the interior lane of the athletic track and were closely followed. The GPS recording started and ended about 15 min before and after the walk	km/h
Leicht and Crowther [44]	Australia	Mean = 20.9 ± 3.8	M = 28 F = 24	150 m on four outdoor surfaces concrete, grass, dry beach sand, wet beach sand	Timed walk	km/h
Murtagh et al. [45]	UK	Range 21–74	M = 28 F = 54	Level, unobstructed path in a public park	Time to walk 18.56 m measured with a stopwatch	m/s
Musselwhite [46]	UK	Mean = 70.5	M = 184 F = 181	Urban shopping area, a suburban residential area and an area of shared space	Speed of walking was captured using software on the trace of the 10-m2 area in real time, when the distance was known	m/s
Noury-Desvaux et al. [47]	France	Experiment 1: Mean = 24 ± 7 Experiment 2: Mean = 32 ± 5	Experiment 1: F = 6 Male = 9 Experiment 1: *N* = 10 (sex NR)	Designated public park (experiment 1) and a 400 m outdoor athletic track (experiment 2)	The actual speed over each bout of walking was calculated by dividing the actual distance travelled by the time measured by chronometry (experiment 2)	km/h
Parise et al. [48]	USA	Range 60–85	M = 117 F = 95	Community walking path	Timed walk. The course consisted of a quarter mile each way (total of half a mile)	km/h
Paysant et al. [49]	France	Mean = 39.7	M = 10	Oval-shaped track. Each subject walked on three ground surfaces: asphalt, mown lawn (grass height ~ 1 in., 2–4 cm maximum), and untended uneven ground (grass height 5 in., 12–20 cm maximum)	The subjects walked on each of the 3 types of terrain for 10 min. Average walking speed was expressed as the time (measured with a chronometer) required for a subject to walk 100 m during the last 2 min of the recording	m/min
Porcari et al. [18]	USA	Range = 30–69	M = 165 F = 178	Measured track—outdoors	1 mile walk	m/s
Prupetkaew et al. [50]	Thailand	Range 18-NR	M = 6 F = 18	A busy 6 m × 16 m × 5 m walkway connected to a parking lot, coffee shop, and was situated along a pedestrian thoroughfare	Participants were asked to walk at their self-selected comfortable walking speed across a 10-m walkway	m/s
Rassafi and Mohajeri [51]	Iran	NR	NR	Pedestrian area	The video footage (captured by traffic control cameras) of pedestrian movement on weekdays from 12.00 p.m. to 06.00 p.m. within a designated area	m/s
Sakazaki et al. [52]	Japan	Range = 55–90 Mean = 68	M = 0 F = 1061	Path	10 m straight one-way path	m/min
Sato et al. [53]	Japan	NR	M = 139 F = 479	Flat, straight path in a residential area of Fukuoka city	The time for each subject to traverse 50 m was measured with a stopwatch during the videotape replay	m/min
Scaglioni-Solano and Aragón-Vargas [16]	Costa Rica	Older adults mean = 69.6 Young adults mean = 22.1	M = 29 F = 28	Outdoor walkways	Timed 15 m walk	m/s
Scaglioni-Solano and Aragón-Vargas [54]	Costa Rica	Women mean = 69.7 Men mean = 71.6	M = 32 F = 90	Flat outdoor walkway	Steps were counted over the 10 m-marked distance	m/s
Silva et al. [55]	Portugal	NR	M = 159 F = 131	Pedestrian footways in eight study locations in the city of Coimbra, Portugal,	Route of approximately 25 m in length was defined. Pedestrian speed was extracted from the video footage, based on distance and time	m/s
Spelman et al. [56]	USA	Mean = 34.9	M = 7 F = 22	Outdoor walking route (participant's usual route)	Time to walk 37 ± 7.6 m level terrain	m/s
Taylor et al. [57]	UK	Mean = 54	M = 3 F = 7	1 km circuit in the university grounds, which had a mixed terrain of concrete and grass)	Global positioning system (GPS) over a 1 km outdoor walk	m/s
Washburn and Laporte [58]	USA	Mean = 28	M = 9 F = 8	.55 mi on sidewalks on the University of Pittsburgh campus	Timed walk	mph
Waters et al. [59]	USA	Range = 20–80	M = 65 F = 81	60.5 m circular outdoor track	When subjects reached a steady state (generally within 3–5 min) and plateaus of heart rate and respiratory rate were observed, a 2-min period of gas collection ensued	m/min
Waters et al. [60]	USA	Range = 20–80	M = 41 F = 70	Level outdoor track 60.5 m in circumference	When they reached a steady state (generally within 3–5 min) and plateaus of heart rate and respiratory rate were observed, a 2-min period of gas collection ensued	m/min
Wettstein et al. [61]	Germany and Israel	Mean = 72.5	M = 73 F = 73	Out of home walking	GPS monitor worn at all times for up to 4 weeks	km/h
Withers et al. [19]	Australia	Range = 55–65 Mean = 59.3	M = 0 F = 50	Level terrain outdoors		km/h

*F* female, *ft/s* feet per second, *GPS* global positioning system, *km/h* kilometres per hour, *M* male, *m* metre, *min* minute, *mph* miles per hour, *m/min* metres per minute, *m/s* metres per second, *NR* not reported,* km* kilometre,* mi* mile

Potential for risk of bias, assessed using the NIH tool for Assessing the Quality of Observational Cohort and Cross-Sectional Studies, is shown in Table 2. No study demonstrated low risk of bias across all domains.

**Table 2:** Assessment of study quality

Study	Research question	Study population	Participation rate	Population and eligibility	Sample size justification	Outcome measures
Abraham et al. [33]	✓	x	O	✓	x	✓
Ali et al. [34]	✓	x	O	✓	x	O
Bargegol et al. [35]	✓	x	O	x	O	O
Bargegol and Gilani [36]	x	x	O	x	O	O
Bassey et al. [22]	✓	x	O	O	x	✓
Braham et al. [37]	✓	x	O	O	x	✓
Brooks et al. [38]	✓	x	O	x	✓	✓
Caramia et al. [39]	✓	x	O	✓	x	✓
Gates et al. [40]	✓	x	O	x	x	x
Gunn et al. [17]	✓	x	O	x	x	✓
Gunn et al. [41]	✓	x	O	O	✓	✓
Gunn et al. [42]	✓	x	O	O	✓	✓
Hills et al. [20]	✓	✓	O	O	x	✓
Le Faucher et al. [43]	✓	x	O	x	x	✓
Leicht and Crowther [44]	✓	x	O	x	x	✓
Murtagh et al. [45]	✓	x	O	x	x	✓
Musselwhite [46]	✓	x	O	x	x	✓
Noury-Desvaux et al. [47]	✓	x	O	x	x	✓
Parise et al. [48]	✓	✓	x	✓	x	✓
Paysant et al. [49]	✓	x	O	O	x	✓
Porcari et al. [18]	✓	x	O	x	x	✓
Prupetkaew et al. [50]	✓	x	O	✓	x	✓
Rassafi and Mohajeri [51]	✓	x	o	O	✓	✓
Sakazaki et al. [52]	✓	✓	O	x	x	✓
Sato et al. [53]	✓	x	O	O	x	✓
Scaglioni-Solano and Aragón-Vargas [16]	✓	x	O	O	x	✓
Scaglioni-Solano and Aragón-Vargas [54]	✓	x	O	x	x	✓
Silva et al. [55]	✓	x	O	x	x	✓
Spelman et al. [56]	✓	x	O	x	x	✓
Taylor et al. [57]	✓	x	O	x	x	✓
Washburn and Laporte [58]	✓	x	O	x	x	✓
Waters et al. [59]	✓	x	O	x	x	✓
Waters et al. [60]	✓	x	O	x	x	✓
Wettstein et al. [61]	✓	x	O	x	x	✓
Withers et al. [19]	✓	x	O	x	x	✓

✓ yes, x no, O other (cannot determine, not applicable, not reported)

**Fig. 2:**
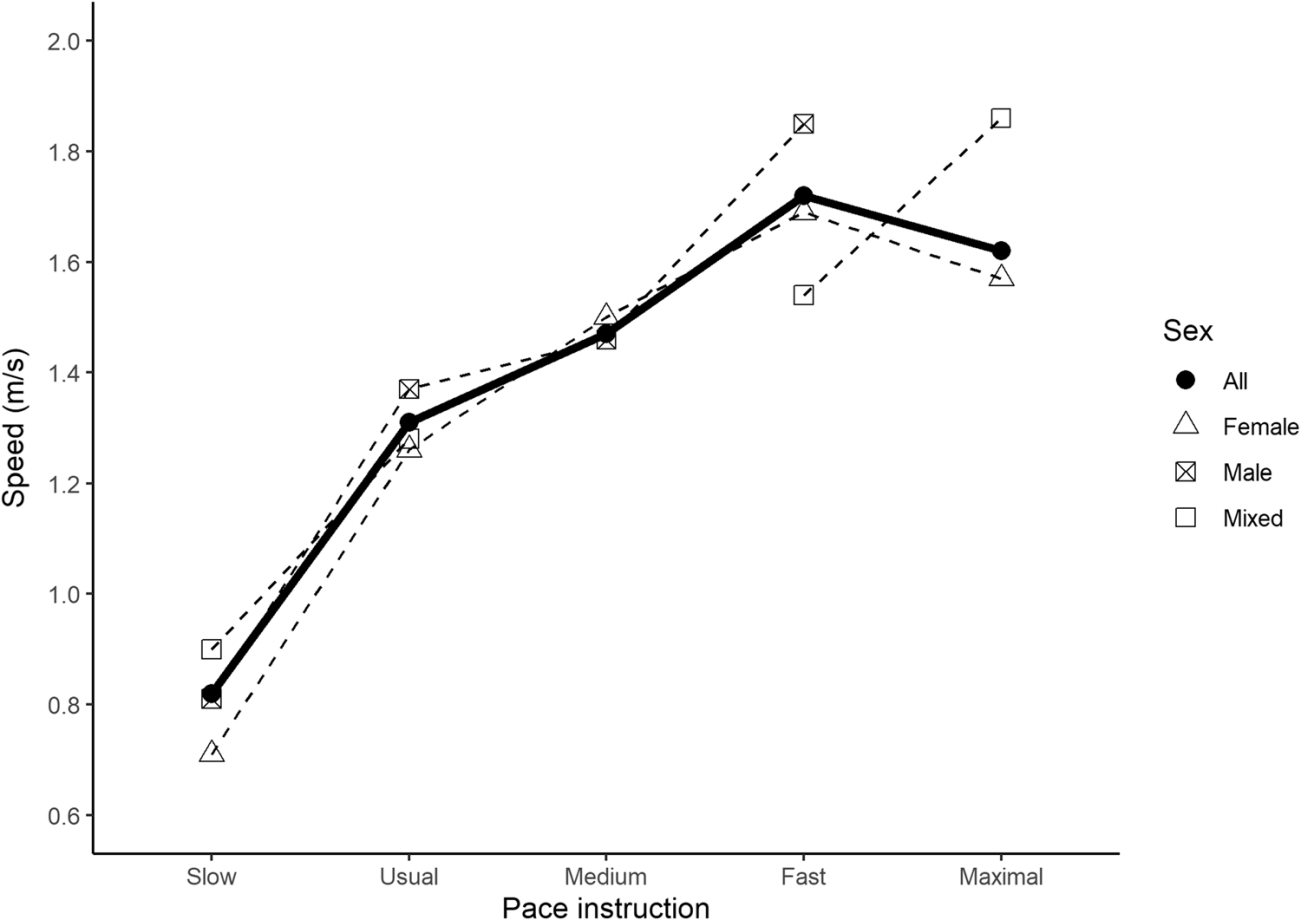
Mean walking speed (m/s). The dashed lines represent the trend for mean speed by pace instruction, separated by sub-group units based on sex (female [triangle], male [square with cross], and mixed sex [open square]). The solid line represents the overall trend for mean speed including all subgroups. Breaks in the lines represent missing data points for that particular sub-group and pace instruction (i.e., mixed medium and maximal males)

**Fig. 3:**
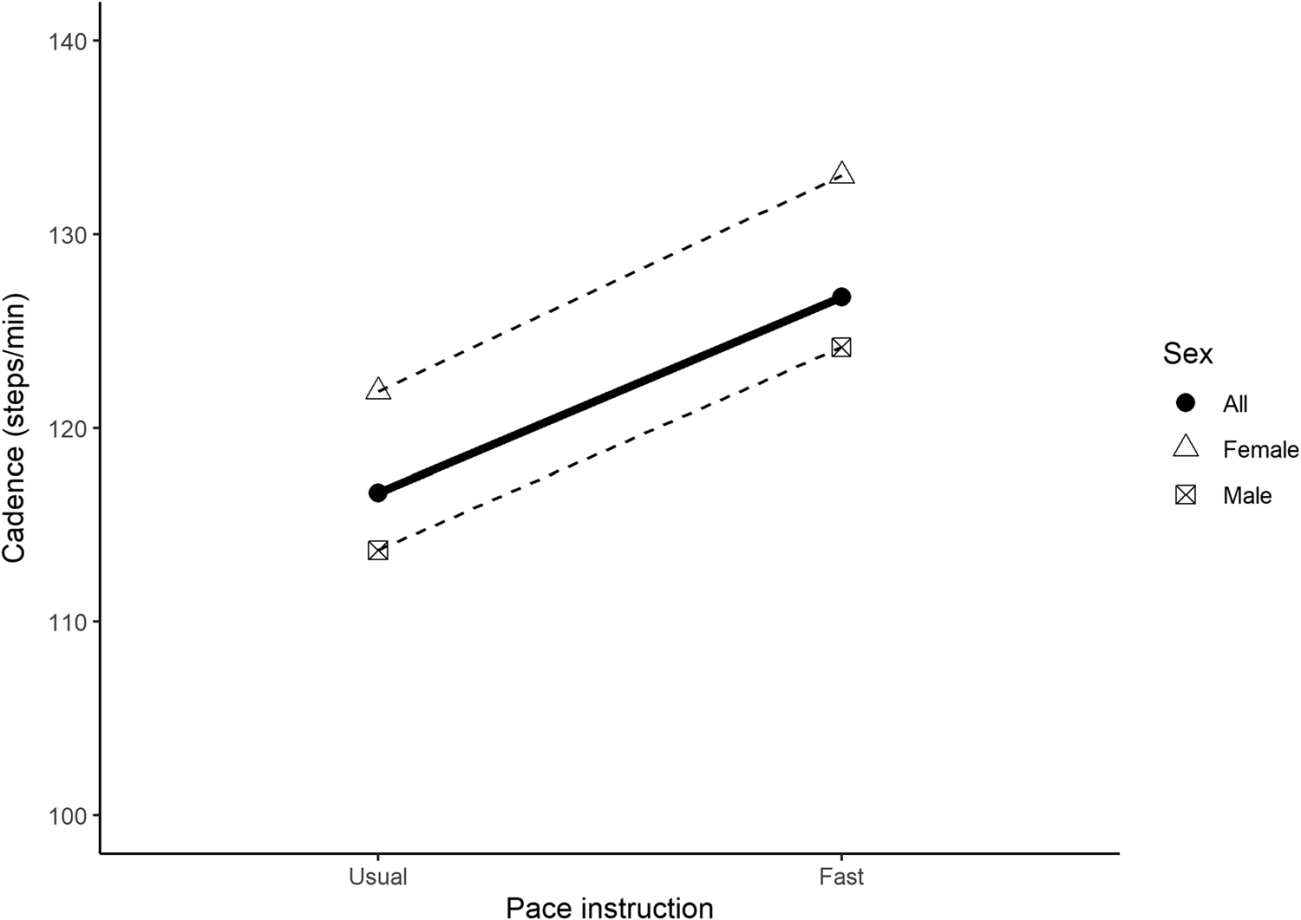
Mean walking cadence (m/s). The dashed lines represent the trend for mean cadence by pace instruction, separated by sub-group units based on sex (female [triangle] and male [square with cross]). The solid line represents the overall trend for mean cadence including all subgroups

Results of the meta-analyses for walking speed according to pace instruction/description category are shown in Table 3. The mean walking speed for slow, usual, medium, fast, and maximal was 0.82 ± 0.02 m/s (9 study groupings, *n* = 201), 1.31 ± 0.02 m/s (111 study groups, *n* = 13,609), 1.47 ± 0.01 m/s (5 study groups, *n* = 208), 1.72 ± 0.05 (19 study groups, *n* = 916), and 1.62 ± 0.09 m/s (7 study groups, *n* = 2172) respectively (Fig. 2). The only sex-specific comparison that could be performed demonstrated that men walked faster than women in response to instruction to walk at a usual pace (*P* = 0.03). Results of the meta-analysis for walking cadence according to pace instruction are presented in Table 4. Mean cadence for usual and fast paces were 116.65 ± 0.87 (45 study groups, *n* = 939) and 126.75 ± 2.49 steps/min (6 study groups, *n* = 268), respectively (Fig. 3). There was a significant difference by sex (*P* < 0.01) for usual walking pace, with women performing higher steps/min than men (121.88 vs 121.88) at this pace.

**Table 3:** Meta-analyses of walking speed (m/s) according to pace instruction/description

Pace instruction	Sex	Study groups	*n*	Mean ± SE	Lower 95% CI	Upper 95% CI	*I*^2^	*P *for sex
Slow								
	Females	2	81	0.71 ± 0.09	0.53	0.90	−	−
	Males	2	65	0.81 ± 0.03	0.76	0.87	−	0.31
	Mixed	5	55	0.90 ± 0.07	0.75	1.04	−	−
	All	9	201	0.82 ± 0.02	0.77	0.86	83.95	−
Usual								
	Females	39	6954	1.26 ± 0.04	1.18	1.34	−	−
	Males	37	4029	1.37 ± 0.03	1.30	1.43	−	**0.03**
	Mixed	35	2626	1.28 ± 0.03	1.21	1.35	−	−
	All	111	13,609	1.31 ± 0.02	1.27	1.35	99.30	−
Medium								
	Females	2	86	1.50 ± 0.03	1.44	1.55	−	−
	Males	3	122	1.46 ± 0.02	1.43	1.49	−	0.18
	Mixed	−	−	−	−	−	−	−
	All	5	208	1.47 ± 0.01	1.44	1.49	47.69	−
Fast								
	Females	8	466	1.69 ± 0.06	1.57	1.80	−	−
	Males	8	357	1.85 ± 0.08	1.69	2.00	−	0.10
	Mixed	3	93	1.54 ± 0.14	1.27	1.82	−	−
	All	19	916	1.72 ± 0.05	1.64	1.81	98.38	−
Maximal								
	Females	5	2122	1.57 ± 0.09	1.38	1.75	−	−
	Males	−	−	−	−	−	−	−
	Mixed	2	50	1.86 ± 0.20	1.47	2.25	−	−
	All	7	2172	1.62 ± 0.09	1.45	1.79	98.95	−

Significant differences between female and male-only study groups are indicated (bold values) in the *P* for sex column

*m/s* metres per second, *n* number of participants, *SE* standard error, *CI* confidence interval, *I*^2^ *I*^2^ statistic, *Females* study groups with only female participants, *Males* study groups with only male participants, *Mixed* study groups with both female and male participants, *All* overall value combining all study groups (i.e., female + male + mixed)

**Table 4:** Meta-analyses of walking cadence (steps/min) according to pace instruction/description

Pace instruction	Sex	Study groups	*n*	Mean ± SE	Lower 95% CI	Upper 95% CI	*I*^2^	*P* for sex
Usual								
	Females	17	533	121.88 ± 1.48	118.99	124.77	–	–
	Males	20	285	113.69 ± 1.34	111.07	116.32	–	< 0.01
	Mixed	8	121	114.22 ± 1.79	110.70	117.74	–	–
	All	45	939	116.65 ± 0.87	114.95	118.35	96.04	–
Fast								
	Females	3	171	133.04 ± 4.61	124.00	142.08	–	–
	Males	3	97	124.16 ± 2.96	118.35	129.96	–	0.10
	Mixed	–	–	–	–	–	–	–
	All	6	268	126.75 ± 2.49	121.87	131.63	99.49	–

*steps/min* steps per minute, *n* number of participants, *SE* standard error, *CI* confidence interval, *I*^*2*^ *I*^2^ statistic, *Females* study groups with only female participants, *Males* study groups with only male participants, *Mixed* study groups with both female and male participants, *All* overall value combining all study groups (i.e., female + male + mixed)

Significant differences between female and male-only study groups are indicated (bold values) in the *P* for sex column

In terms of absolute intensity, participants walked at 4.21 ± 0.14 METs (medium pace, 7 study groupings, *n* = 232). Relative intensity for a medium pace was 55.86 ± 0.89%HR_max_, (4 study groups, *n* = 158). There was a significant difference between women and men, with women exhibiting a higher percentage HR_max_ than men (61.0 vs 53.1%HR_max_; see Table 5).

**Table 5:** Meta-analyses of intensity outcomes according to pace instruction/description

Outcome and pace instruction	Sex	Study groups	*n*	Mean ± SE	Lower 95% CI	Upper 95% CI	*I*^2^	*P* for sex
METs								
Medium								
	Females	3	98	4.14 ± 0.18	3.79	4.48	–	–
	Males	4	134	4.36 ± 0.26	3.85	4.86	–	0.48
	Mixed	–	–	–	–	–	–	–
	All	7	232	4.21 ± 0.14	3.92	4.49	91.70	–
%HR_max_								
Medium								
	Females	1	36	61.00 ± 1.50	58.06	63.94	–	–
	Males	3	122	53.09 ± 1.10	50.93	55.25	–	** < 0.01**
	Mixed	–	–	–	–	–	–	–
	All	4	158	55.86 ± 0.89	54.12	57.60	89.55	–

Significant differences between female and male-only study groups are indicated (bold values) in the *P* for sex column

*n* number of participants, *SE* standard error, *CI* confidence interval, *I*^*2*^ *I*^2^ statistic, *METs* metabolic equivalents, *%HR*_*max*_ percentage maximal heart rate, *Females* study groups with only female participants, *Males* study groups with only male participants, *Mixed* study groups with both female and male participants, *All* overall value combining all study groups (i.e., female + male + mixed)

**Fig. 4:**
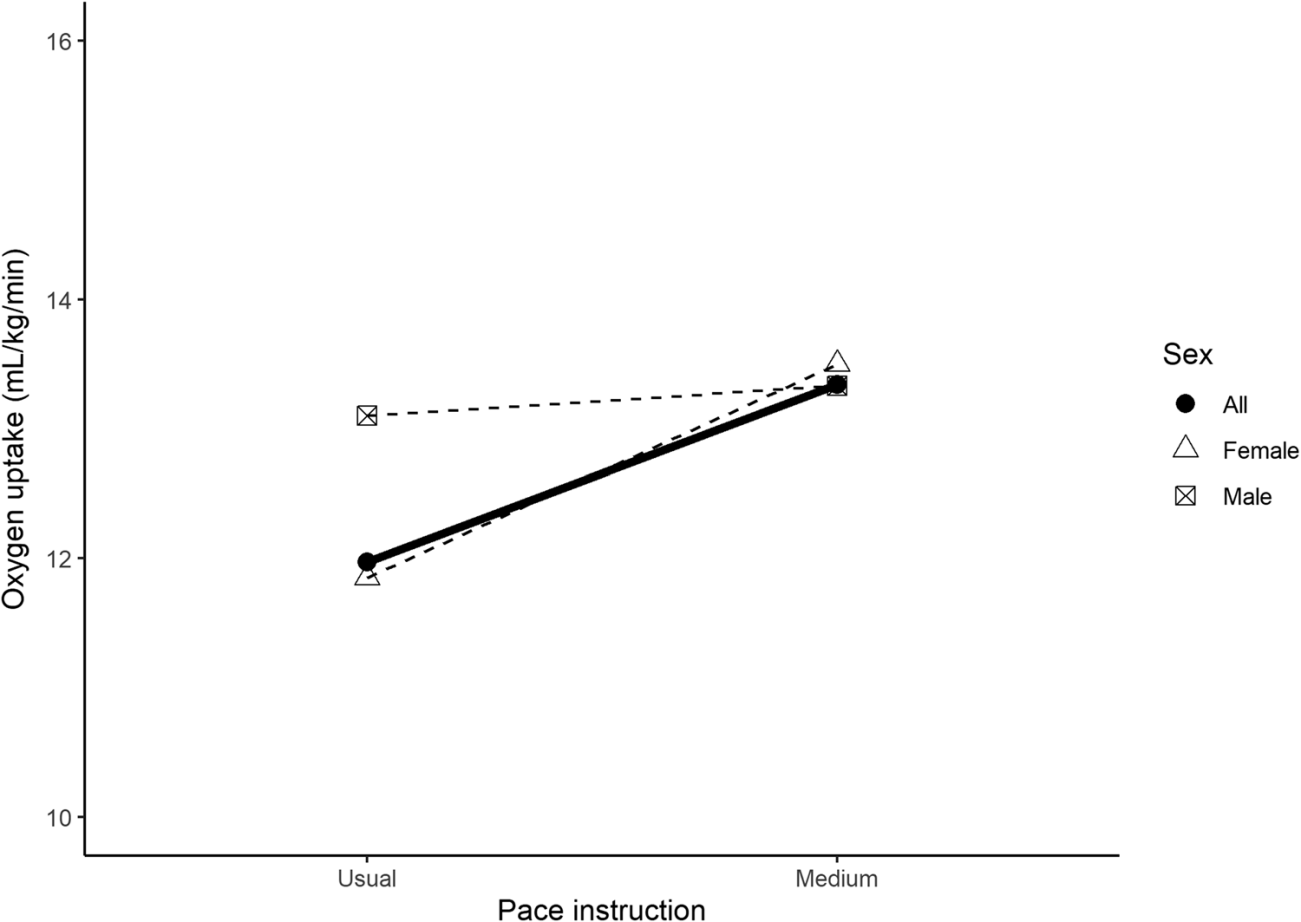
Mean walking oxygen uptake (mL/kg/min). The dashed lines represent the trend for mean oxygen uptake by pace instruction, separated by sub-group units based on sex (female [triangle], male [square with cross]). The solid line represents the overall trend for mean oxygen uptake including all subgroups

Table 6 shows findings for meta-analyses of oxygen consumption and energy expenditure outcomes according to pace instruction. The mean oxygen consumption for the usual and medium pace instructions was 11.97 ± 0.14 mL/kg/min (11 study groups, *n* = 287) and 13.34 ± 0.20 mL/kg/min (4 study groups, *n* = 110), respectively (Fig. 4). There was a significant difference between women and men for the usual pace instruction (*P* = 0.01), with men demonstrating a higher oxygen consumption than women (13.1 vs 11.85 mL/kg/min). The mean energy expenditure in response to a medium pace instruction was 1229.3 ± 24.93 kJ/h (4 study groups, *n* = 96) and 16.17 ± 0.25 kJ/kg/h (4 study groups, *n* = 110).

**Table 6:** Meta-analyses of oxygen consumption and energy expenditure outcomes according to pace instruction/description

Outcome and pace instruction	Sex	Study groups	*n*	Mean ± SE	Lower 95% CI	Upper 95% CI	*I*^2^	*P* for sex
mL/kg/min								
Usual								
	Females	4	151	11.85 ± 0.15	11.56	12.15	–	–
	Males	7	136	13.10 ± 0.47	12.18	14.03	–	**0.01**
	Mixed	–	–	–	–	–	–	–
	All	11	287	11.97 ± 0.14	11.69	12.25	99.31	–
Medium								
	Females	1	12	13.50 ± 0.98	11.58	15.42	–	–
	Males	3	98	13.33 ± 0.20	12.93	13.73	–	0.86
	Mixed	–	–	–	–	–	–	–
	All	4	110	13.34 ± 0.20	12.94	13.73	0.00	–
kJ/h								
Medium								
	Females	2	48	1153.33 ± 32.83	1088.97	1217.68	–	–
	Males	2	48	1332.68 ± 38.30	1257.61	1407.74	–	** < 0.01**
	Mixed	–	–	–	–	–	–	–
	All	4	96	1229.30 ± 24.93	1180.44	1278.16	77.91	–
kJ/kg/h								
Medium								
	Females	1	12	16.50 ± 1.27	14.01	18.99	–	–
	Males	3	98	16.16 ± 0.25	15.67	16.65	–	0.79
	Mixed	–	–	–	–	–	–	–
	All	4	110	16.17 ± 0.25	15.69	16.66	0.00	–

Significant differences between female and male-only study groups are indicated (bold values) in the *P* for sex column

*Females* study groups with only female participants, *Males* study groups with only male participants, *Mixed* study groups with both female and male participants, *All* overall value combining all study groups (i.e., female + male + mixed)

## Discussion

This is the first systematic review and meta-analysis to aggregate the results of comparable measures of outdoor walking speed and intensity indicators extracted from methodologically and regionally diverse studies representing more than 14,000 research participants and 13 countries. We demonstrate that outdoor walking speed, cadence, intensity, and energy expenditure increase commensurate with more challenging pace instructions. This systematic review provides the first summary of typical outdoor walking speeds enacted for a range of pace instructions. This summary provides evidence-based reference values against which data from other individuals or future studies can be compared.

The “Compendium of Physical Activities” is widely used as a resource to estimate and classify the energy cost of human physical activity [24]. We determined that an instruction to walk at a slow pace was associated with 0.82 m/s (95% CI 0.77–0.86). This aligns with the Compendium’s activity code 17151, listing walking “less than 0.89 m/s, level, strolling, very slow” as 2.0 METs. Walking speed associated with usual pace instruction was 1.31 m/s (95% CI 1.27–1.35), aligning with Compendium activity code 17190 (“walking, 1.25–1.43 m/s, level, moderate pace, firm surface”) and 3.5 METs. Walking speed associated with a medium pace instruction (1.47 m/s, 95% CI 1.44–1.49) was slightly faster than this Compendium code’s definition. The upper 95% confidence interval of the walking speed associated with a fast pace instruction (1.72 m/s, 95% CI 1.64–1.81) reached the Compendium’s threshold for 5.0 METs. Using the commonly used definition of moderate intensity as 3.0–5.9 METs [25], we estimate that, with the exception of the slow pace instruction, all other pace (i.e. usual, medium, fast, and maximal) instructions provided in the assembled studies would have elicited a walking intensity within a range associated with absolutely defined moderate intensity. This is important as public health guidelines recommend that adults should accumulate at least moderate-intensity aerobic activity [26] to accrue optimal health benefit.

Of note is that the mean walking speed for the maximal pace instruction/description category was lower than the fast category (1.62 ± 0.09 m/s vs 1.72 ± 0.05). This unusual finding could be because the maximal walking speed test is commonly conducted in older adults—who demonstrate lower maximal walking speed than younger adults—however, as sub-group analysis by age was not possible, this explanation is speculative. The overwhelming majority of participants in studies contributing data to the maximal category were female. Sex differences in walking speed have previously been demonstrated, with men attaining higher speeds in short-distance walking tests than women due to differences in height and leg length [27, 28].

Studies that include measures of absolute intensity (e.g., METs) and/or relative intensity (e.g., percentage maximal heart rate) can offer a more direct indication of whether or not a specific walking speed can reach public health moderate-intensity guidelines. There is a strong relationship between cadence and intensity, with > 100 steps/min established as a threshold value associated with absolutely defined moderate intensity [29]. For both the usual pace and fast pace categories, steps/min exceeded this threshold, with the fast pace averaging 126.8 steps/min (95% CI 121.9–131.6). Similarly, the mean METs observed for the medium pace instruction of 4.2 METs (95% CI 3.9–4.5) are concordant with the commonly used definition of moderate intensity of 3.0–5.9 METs [25]. However, in terms of common definitions of relative exercise intensity, the medium pace instruction only elicited a heart rate response 55.9% HR_max_ (95% CI 54.1–57.6) that would be considered only very light intensity [25]. Moderate intensity is considered to be 64–79% HR_max_ [25]. Our finding that medium pace instruction only evoked light intensity may be due to the age and fitness of participants. Maximum heart rate declines with age. We note that both of the three studies from which these groups—providing data on percentage maximal heart rate in response to the medium pace instruction—are drawn reported a mean age of 40 years. These younger individuals with a higher maximal heart rate will need to walk at a faster pace to reach 64% of their HR_max_ compared to older individuals with a lower maximum. Furthermore, within any age group, individuals with higher levels of cardiorespiratory fitness (and therefore larger cardiac output) will have less elevation in HR at a given speed than those with lower levels of fitness.

We note that a greater relative exercise intensity was observed for females (61.0%HR_max_) compared to males (53.1%HR_max_) in response to the medium pace instruction. This gender difference may be explained by the known lower cardiac output (due to smaller heart size) and lean body mass of females compared to males at a given age [30]

### Limitations

Several limitations of this systematic review and meta-analysis are noted. First, our assessment of study quality noted that while the research question and outcomes measures were clearly stated in nearly all studies, many failed to clearly specify the study population. For example, often only subject numbers and location were reported. There is the possibility of selection bias in some studies. Also, in most cases, a sample size justification, power description, or variance and effect estimates were not reported in the original study. These threats to internal validity may increase the risk for bias. Second, the purpose of walking was not considered (e.g., for commuting and for recreation) but may theoretically have an effect on enacted walking speed. Third, a variety of tests/protocols were used to measure walking pace, and therefore across studies, there is no standardised assessment method. Fourth, there were not sufficient data available on age to permit sub-group analysis. Fifth, as we restricted our search to articles published in English, it is possible that there are additional studies published in other languages that could augment this evidence base. Finally, studies undertaken in Australia and the US contributed 23% and 20% of all included studies; therefore, findings may not be representative of broader populations. Our assessment of study quality indicates the need for better designed and executed studies, although it is possible that they only neglected to report on assessed items. We note that several guidelines are gaining prominence which aims to enhance standardised reporting of observational studies (e.g., the STROBE statement [31]). Adherence to such guidelines should enhance the secondary use and analysis of data.

### Implications for Research

Our analyses highlight participant subgroups where data were not available. For example, no cadence data were available for slow or medium paces. Furthermore, we could only present relative intensity findings in terms of %HR_max_ and only specifically for medium pace instruction. In addition, due to a lack of data, we were unable to provide any age-specific expected values. These gaps in the evidence base should be addressed in future studies.

Findings on outdoor walking pace would be enhanced with standardised data collection that includes participants’ height, BMI, and leg length, as these are known to influence gait speed [32]. However, we acknowledge that for studies that observe “real world” walking speed in outdoor settings, it is difficult to always capture these data. A trade-off must be made between observation of large numbers of individuals (thus enhancing the precision of results) and collecting anthropometric data on each individual (thereby enhancing the applicability of findings to specific subgroups).

## Conclusion

As noted above, walking speed is related to intensity. In a controlled setting, it is easier to set a speed (e.g., on a treadmill) to elicit a desired intensity level. Without relying on more advanced wearable technologies, it is more challenging to elicit a specific intensity, let alone a specific speed, without clear instruction. The amalgamated data herein provide expected values (where available) for speed, cadence, percent maximal heart rage, and oxygen consumption for slow through maximal paced walking. Walking at a self-selected (usual) pace was associated with an average speed of 1.31 m/s, a cadence of 116.65 steps/min, and an oxygen consumption of 11.97 mL/kg/min, meeting/exceeding public health thresholds for moderate-intensity activity The assembled information provides greater clarity with regard to how various indicators of enacted walking pace, speed, and intensity overlap and how each can be best communicated in the real-world setting to optimise health-related outcomes. Pace-based instructions can be used to support walking in outdoor settings within public health guidelines.

## Electronic supplementary material

Below is the link to the electronic supplementary material.

Supplementary file1 (DOCX 15 kb)

## Declarations

### Data Availability Statement

The dataset generated during the current review is available in the OSF repository, https://osf.io/uypx6/?view_only=e7b6071fc2ec4e04b1140620fc716e51.
