# Supplementary material for: Outdoor Walking Speeds of Apparently Healthy Adults: A Systematic Review and Meta-analysis
Source: Sports Med. 2020 Oct 8;51(1):125–41. doi: 10.1007/s40279-020-01351-3 (PMC7806575; doi:10.1007/s40279-020-01351-3)
Supplement: Supplementary file 1 — Supplementary file1 (DOCX 15 kb) [file 40279_2020_1351_MOESM1_ESM.docx]

**Electronic Supplementary Material Appendix S1: Search Strategy**

**Search Strategy**

| 1 | Walking [mh] |
| --- | --- |
| 2  3 | Exp Gait [mh]  Ambulat* OR gait* OR stride OR walk* [mp] |
| 4 | 1 OR 2 OR 3 |
| 5 | Acceleration (Physiology) [mh] |
| 6 | Accelerat* OR speed* OR velocity [mp] |
| 7 | 5 OR 6 |
| 8 | Normal OR comfort* OR self-selected OR usual OR prefer* [mp] |
| 9  10  11 | 4 AND 7 AND 8  Exp Adult [mh]  9 AND 10 |

[mp=title, abstract, original title, name of substance word, subject heading word, keyword heading word, protocol supplementary concept word, rare disease supplementary concept word, unique identifier, synonyms]

[mh=MESH]
